# Late vertebral side effects in long-term survivors of irradiated childhood brain tumor

**DOI:** 10.1371/journal.pone.0209193

**Published:** 2018-12-18

**Authors:** Miro-Pekka Jussila, Tiina Remes, Julia Anttonen, Arja Harila-Saari, Jaakko Niinimäki, Tytti Pokka, Päivi Koskenkorva, Anna Sutela, Sanna Toiviainen-Salo, Pekka Arikoski, Pekka Riikonen, Mikko Arola, Päivi Lähteenmäki, Kirsti Sirkiä, Heikki Rantala, Maria Suo-Palosaari, Marja Ojaniemi

**Affiliations:** 1 Research Unit of Medical Imaging, Physics and Technology, Faculty of Medicine, University of Oulu, Oulu, Finland; 2 Medical Research Center, University of Oulu, Oulu, Finland; 3 Department of Pediatrics and Adolescence, Oulu University Hospital, Oulu, Finland; 4 PEDEGO Research Unit, Oulu University Hospital and University of Oulu, Oulu, Finland; 5 Department of Women’s and Children’s Health, Uppsala University, Uppsala, Sweden; 6 Department of Diagnostic Radiology, Oulu University Hospital and University of Oulu, Oulu, Finland; 7 Department of Clinical Radiology, Kuopio University Hospital, Kuopio, Finland; 8 Department of Pediatric Radiology, HUS Medical Imaging Center, Radiology, University of Helsinki and Helsinki University Hospital, Helsinki, Finland; 9 Department of Pediatrics and Adolescence, Kuopio University Hospital and University of Eastern Finland, Kuopio, Finland; 10 Department of Pediatrics, Tampere University Hospital and University of Tampere, Faculty of Medicine and Life Sciences, Tampere, Finland; 11 Department of Pediatrics and Adolescent Medicine, Turku University Hospital and Turku University, Turku, Finland; 12 Department of Pediatrics and Adolescence, Helsinki University Hospital, Helsinki, Finland; Medical College of Wisconsin, UNITED STATES

## Abstract

**Purpose:**

Long-term side effects of the treatments are common in survivors of irradiated pediatric brain tumors. Ionizing radiation in combination with surgery and chemotherapy during childhood may reduce vertebral height and bone mineral density (BMD), and cause growth failure. The aim of this study was to evaluate the late consequences of tumor treatments on vertebrae in survivors of childhood brain tumors.

**Methods:**

72 adult survivors (mean age 27.8 years, standard deviation 6.7) of irradiated childhood brain tumor were studied by spinal magnetic resonance imaging (MRI) for vertebral abnormalities from the national cohort of Finland. Patients were treated in five university hospitals in Finland between the years 1970 and 2008. Subject height and weight were measured and body mass index (BMI) was calculated. The morphology and height/depth ratio of the vertebrae in the middle of the kyphotic thoracic curvature (Th8) and lumbar lordosis (L3) were examined. Vertebrae were analyzed by Genant’s semiquantative (SQ) method and spinal deformity index (SDI) was calculated. BMD was measured by using dual X-ray absorptiometry.

**Results:**

4.2% (3/72) of the patients had undiagnosed asymptomatic vertebral fracture and 5.6% (4/72) of patients had radiation-induced decreased vertebral body height. Male patients had flatter vertebrae compared with females. Patient age at the time of irradiation, BMI and irradiation area correlated to vertebral morphology differentially in males and females. BMD had no association with the vertebral shape. Patients who had received craniospinal irradiation were shorter than the general population.

**Conclusion:**

Childhood brain tumor survivors had a high number of vertebral abnormalities in young adulthood. Irradiation was associated with abnormal vertebral morphology and compromised final height. Male gender may predispose vertebrae to the side effects of irradiation.

## Introduction

Central nervous system tumors are the most common solid tumors in children [1,2], and they account for 26% of all pediatric cancers [3]. The mortality rate for brain tumors has decreased over recent decades due to more effective cancer treatments [1], resulting in a growing adult population of childhood brain tumor survivors. Treatment options for brain tumors include surgical operations, chemotherapy and radiation therapy [4].

The most common late effects of childhood brain tumors and their treatments are neuropsychological and endocrinological disturbances [4]. In addition, other known late effects include cardiovascular diseases, reduced fertility, secondary malignancies, specific organ toxicities and delayed growth [1,4,5]. A brain tumor itself, radiation therapy and other treatments may affect bone health [5–9]. Radiation therapy can directly cause bone destruction [6,10], and craniospinal irradiation has been shown to affect patient growth [11,12]. The lumbar vertebrae are more prone to growth impairment caused by irradiation than cervical or thoracic vertebrae [11]. The wider the irradiated area of the spine is, the stronger the impact on the patient height [13]. The signal intensity of the vertebrae has been shown to be heterogeneous and high on T1-weighted (T1W) images after craniospinal irradiation, suggesting fatty replacement of spinal bone marrow [13].

This study was carried out to evaluate the late effects on vertebrae in survivors of irradiated childhood brain tumor. For this, a national cohort of irradiated brain tumor survivors was gathered, and the vertebral bone health of the subjects was systematically analyzed.

## Methods

### Study population

**Fig 1:**
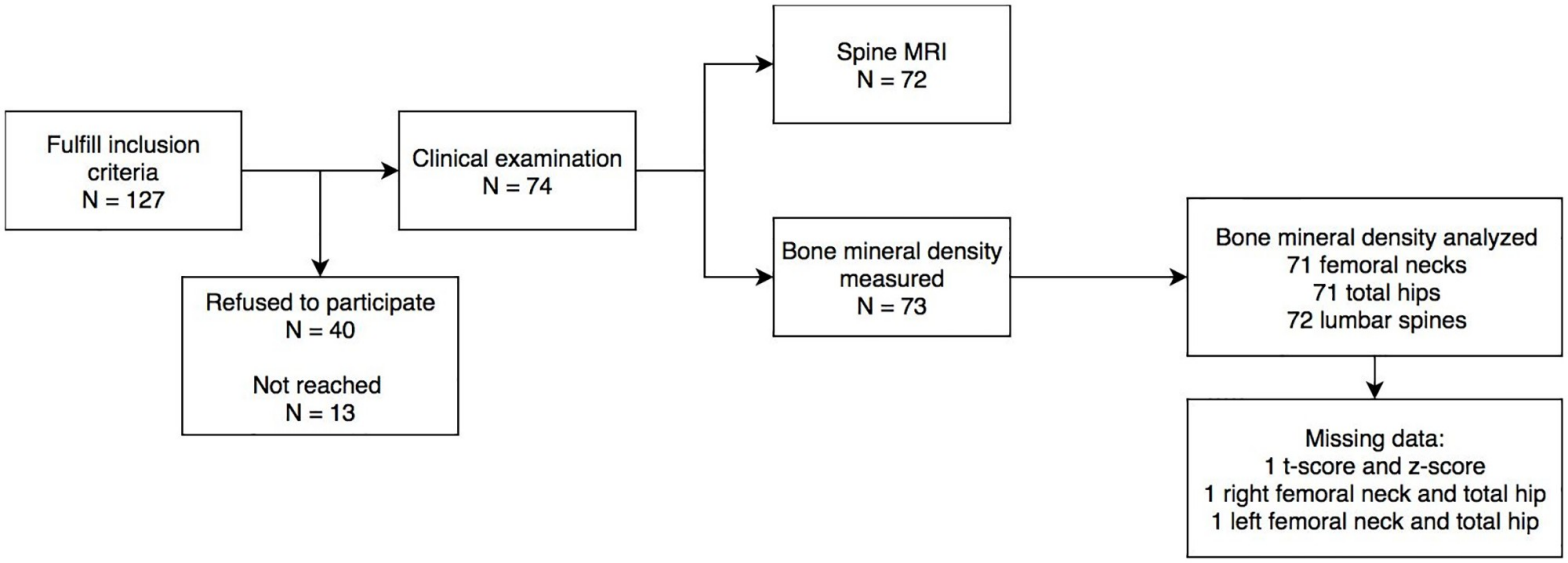
Flowchart of the study subjects.

The study population consisted of patients who had been treated for brain tumors in childhood between the years 1970 and 2008. Brain tumors were diagnosed before the age of 16 years. The treatment of the brain tumors included surgery, irradiation and/or chemotherapy. The therapies had ended a minimum of 5 years before this study started. All subjects were at least 16 years of age and no known progressive diseases had been diagnosed at the time of the present study. Patients were treated at the University Hospitals of Oulu, Kuopio, Turku, Tampere or Helsinki. Clinical examinations were done on 74 of the 127 invited subjects and spinal magnetic resonance imaging (MRI) on 72 of the 74 clinically examined subjects (Fig 1).

### Spinal MRI analyses

Spinal MRI was done to 72 subjects during the years 2010–2015. MRI scans were done with a Siemens Magnetom Espree 1.5T scanner at Oulu University Hospital, Siemens Avanto 1.5T scanners at Helsinki, Kuopio and Tampere University Hospitals, and a Philips Ingenia 1.5T scanner at Turku University Hospital. The spinal MRI protocol included contrast-enhanced T1-weighted (T1W) spin echo (SE) sagittal and T2-weighted (T2W) SE sagittal sequences. Gadolinium contrast agent (Dotarem, 0.2 ml/kg, Guerbet, France) was used. MRI scans were evaluated on a research PACS/DICOM viewing application for diagnostic radiology (neaView, Neagen, Helsinki, Finland). The shape of the vertebrae was evaluated and identified for possible fractures. Vertebrae from the fourth thoracic to the fourth lumbar vertebra were analyzed according to Genant’s visual semiquantitative (SQ) method [14,15]. A spinal deformity index (SDI) was calculated by summing the grades of vertebral deformities [14]. Primary and secondary tumors of the spine were excluded. To analyze the morphology of the thoracic and lumbar vertebrae more specifically, the height and depth of one thoracic vertebra (Th8) in the middle of kyphotic thoracic curvature and one lumbar vertebra (L3) in the middle of lordotic lumbar curvature were measured. The height of the Th8 and L3 vertebrae was measured from the lowest part of the vertebral body. The depth (anterior-posterior measurement) of the Th8 and L3 vertebral bodies was measured from the superior and inferior end plates. The height/depth ratio of these vertebrae was calculated.

### Clinical examination

Clinical examination was done to 74 participants by a pediatric neurologist (author TR). A questionnaire was used to gather information about previous fractures. The tumor treatment, including corticosteroid medication, was studied from the patient files. Subject height and weight were measured and body mass index (BMI) was calculated. The standard deviation score (SDS) of the height of each subject was obtained by using the new Finnish growth standard [16]. All subjects except one were capable of moving without any walking aid.

### Densitometry

Subjects were examined using dual X-ray absorptiometry (DXA). Here, the results from our previously published DXA analyses [9] were compared with the spinal MRI findings. As described earlier, the bone mineral content and bone mineral density (BMD) of the lumbar spine and four femoral sites (femoral necks and total hips) were measured by using DXA with Lunar Prodigy DXA bone densitometry at Oulu, Lunar Prodigy Advance DXA bone densitometry at Kuopio, Lunar iDXA DXA densitometry at Tampere (Lunar Corporation, General Electric Madison, WI, USA), Hologic Discovery A DXA at Helsinki and Hologic QDR 4500C DXA densitometry at Turku (Hologic Inc., Bedford, MA, USA). The results were expressed as age- and gender-normalized z-scores provided by the manufacturers. The International Society for Clinical Densitometry recommends reporting BMD in premenopausal women and in men < 50 years of age as z-scores (BMDZ) [17]. A z-score of -2.0 indicates a result below the expected range for age and gender [17]. Thus, two BMD groups were formed, z-score >-2.0 group and z-score ≤ -2.0 group.

### Statistics

The analysis of variance (ANOVA) was used to determine whether there were any statistically significant differences between the means of more than two groups. A non-parametric Kruskal-Wallis test was used when the variables were not normally distributed. The association of tumor treatments (irradiation, chemotherapy and total dose of corticosteroids administered during brain tumor treatment), BMI and treatment age to vertebra height/depth ratio was examined with multivariate linear regression model with a stepwise variable selection procedure to identify the set of the variables that best predicted vertebral morphology. Spearman correlation coefficient was used when evaluating correlation between corticosteroid dose to the vertebra. Subjects were analyzed in three age groups (0–6, 7–11 and 12–16 years) taking into account different growth periods at the time of the radiation therapy. For all tests, a significance level of < 0.05 was used. All data were analyzed using IBM SPSS statistic 25.0 software (Armonk, NY: IBM Corp.).

### Ethics

Written informed consent was obtained from all the participants included in this study and/or their legal guardians. The Research Ethics Committees of the Northern Ostrobothnia Hospital District, Northern Savo Hospital District, Southwest Finland Hospital District, Pirkanmaa Hospital District and Helsinki and Uusimaa Hospital District approved the study. The research was in accordance with the principles of the Declaration of Helsinki.

## Results

### Patient characteristics

The cohort has been described in detail by Remes et al. [9]. The most common tumors of the patient cohort are shown in Table 1. Males accounted for 63.5% (*n* = 47) of the subjects and females 36.5% (*n* = 27). Surgical operations were done in 82.4% of the cases. In 10 cases, a biopsy only was taken, and in three cases, no surgical procedures were done. A total tumor resection was done in 29 and partial resection in 32 of the cases. No primary or secondary tumors of the spine were found.

**Table 1:** Patient characteristics and tumor types.

Patient gender, *n* (%)	male	47 (63.5)
	female	27 (36.5)
Tumor type, *n* (%)	Astrocytoma	24 (32.4)
	Medulloblastoma	20 (27.0)
	Ependymoma	8 (10.8)
	Germinoma	7 (9.5)
	Other	15 (20.3)
Age at diagnosis, yr, mean (SD)	male	7.7 (4.3)
	female	8.2 (4.3)
Age at irradiation, yr, mean (SD)	male	8.1 (4.1)
	female	8.7 (4.0)
Craniospinal irradiation, *n* (%)	yes	30 (40.5)
	no	44 (59.5)
Chemotherapy, *n* (%)	yes	47 (63.5)
	no	27 (36.5)
Age at the study MRI, yr, mean (SD)	male	26.8 (6.1)
	female	29.4 (7.4)

yr = years, MRI = magnetic resonance imaging, SD = standard deviation

All patients had received irradiation: 40.5% (*n* = 30 of 74) of the patients had received craniospinal irradiation, and cranial irradiation only was done to 59.5% (*n* = 44 of 74) patients. Of those who had received cranial irradiation (*n* = 44), whole brain irradiation was done to 6.8% (*n* = 3 of 44) of the patients, local tumor irradiation was done to 88.6% (n = 39 of 44) of the patients and stereotactic irradiation was done to 4.5% (*n* = 2 of 44) of the patients. Chemotherapy was used in 63.5% (*n* = 47 of 74) of the cases: 90% (*n* = 27 of 30) of the patients treated with craniospinal irradiation and 45.5% (*n* = 20 of 44) of the patients treated with cranial irradiation only had chemotherapy.

There were 65 patients who received corticosteroid therapy. The median cumulative dose as prednisolone equivalent was 2.3 g/m^2^ (IQR = 0.9–7.0). The median dose was higher in those patients who had received chemotherapy vs patients who had not [(3.7 g/m^2^, IQR = 1.5–8.2 vs 1.1g/m^2^, IQR = 0.6–1.5), *p* < 0.001]. Corticosteroid doses varied between age groups (*p* = 0.001). The median corticosteroid dose was highest in the youngest age group (0–6 years) (5.7 g/m^2^) and lowest in oldest age group (12–16 years) (1.0 g/m^2^).

### Vertebral fractures and end-plate irregularities

**Fig 2:**
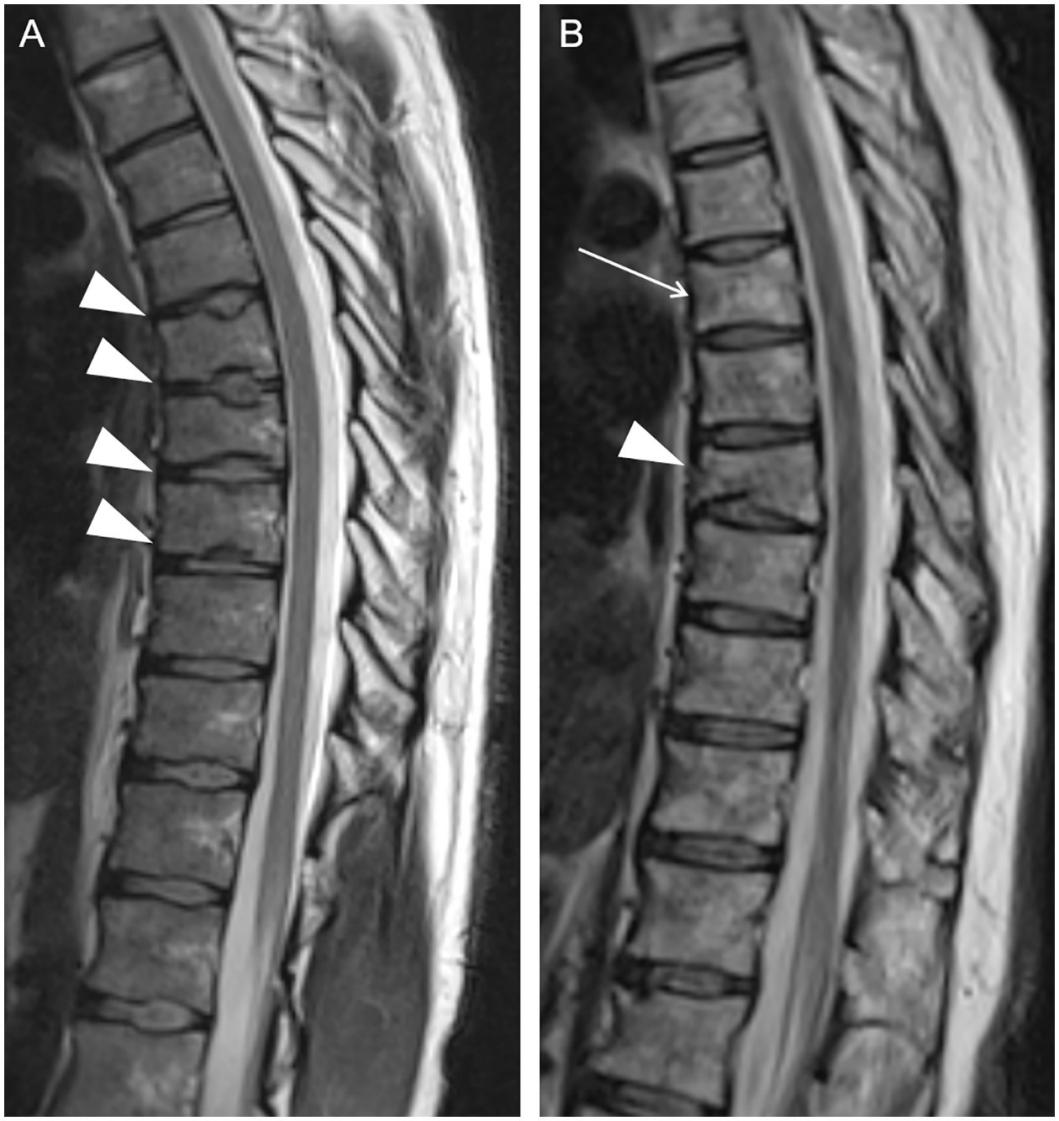
Representative spinal magnetic resonance imaging (MRI) showing vertebral changes on T2-weighted (T2W) sagittal images. Several adjacent flat thoracic vertebrae with end-plate irregularities are demonstrated on T2W image of a male aged 31 years (A, arrowheads). T2W image of a female aged 43 years demonstrates slightly compressed Th6 vertebra, Genant’s grade 1 (B, arrow) and vertebral fracture of Th8, Genant’s grade 2 (B, arrowhead).

In 18 subjects, SDI was greater than 0, indicating vertebral deformity. In 11 of the 18 cases, there were only borderline deformed vertebrae (SDI = 0.5) that were not considered to be definite fractures. When considering only the vertebrae with Genant’s grade ≥ 1 [14], seven of the 18 subjects had at least one vertebra meeting the criteria of osteoporotic fracture. However, four of these subjects had decreased vertebral body height and end-plate irregularities in several adjacent thoracic vertebrae not representing fractures (Fig 2A) but other radiation-induced changes in the spine [13]. Thus, there were three definite cases of vertebral fracture of Genant’s grade ≥ 1, shown in the representative image Fig 2B. The three subjects with vertebral fractures were the oldest patients in this study. Details of these three subjects and their tumor treatments are shown in Table 2. The height of the cervical vertebrae was normal. There were no fractures or morphological changes in cervical vertebrae of the study subjects.

**Table 2:** Description of the subjects who had vertebral fractures.

Patient gender	Genant’s grade	Patient age (yr) at the time of spinal MRI	Corticosteroid dose g/m^2^	Chemotherapy	Craniospinal irradiation	Z-score ≤ -2.0	Previous number of fractures in long bones
male	1	43	1.72	yes	no	no	1
male	1	41	7.98	no	no	yes	1
female	2	43	2.28	yes	yes	no	4

yr = year, MRI = magnetic resonance imaging

### Vertebral morphology

To evaluate the changes of the vertebral morphology, height/depth ratio was calculated from the Th8 and L3 vertebrae. Females had greater mean in height/depth ratio compared with males of both Th8 and L3 vertebrae. Mean values of Th8 and L3 height/depth ratio are presented in Table 3.

**Table 3:** Mean values of Th8 and L3 vertebrae measurements in males and females.

MRI results	All *n* = 72	Male *n* = 46	Female *n* = 26	*p*-value
	Mean (SD)	Mean (SD)	Mean (SD)	
Th8 height/depth ratio %	67.3 (9.0)	65.7 (9.0)	70.2 (8.4)	0.041
L3 height/depth ratio %	75.3 (9.9)	73.3 (10.0)	78.9 (8.9)	0.019

SD = standard deviation, Th = thoracic, L = lumbar

#### Morphology of the Th8 vertebra

**Fig 3:**
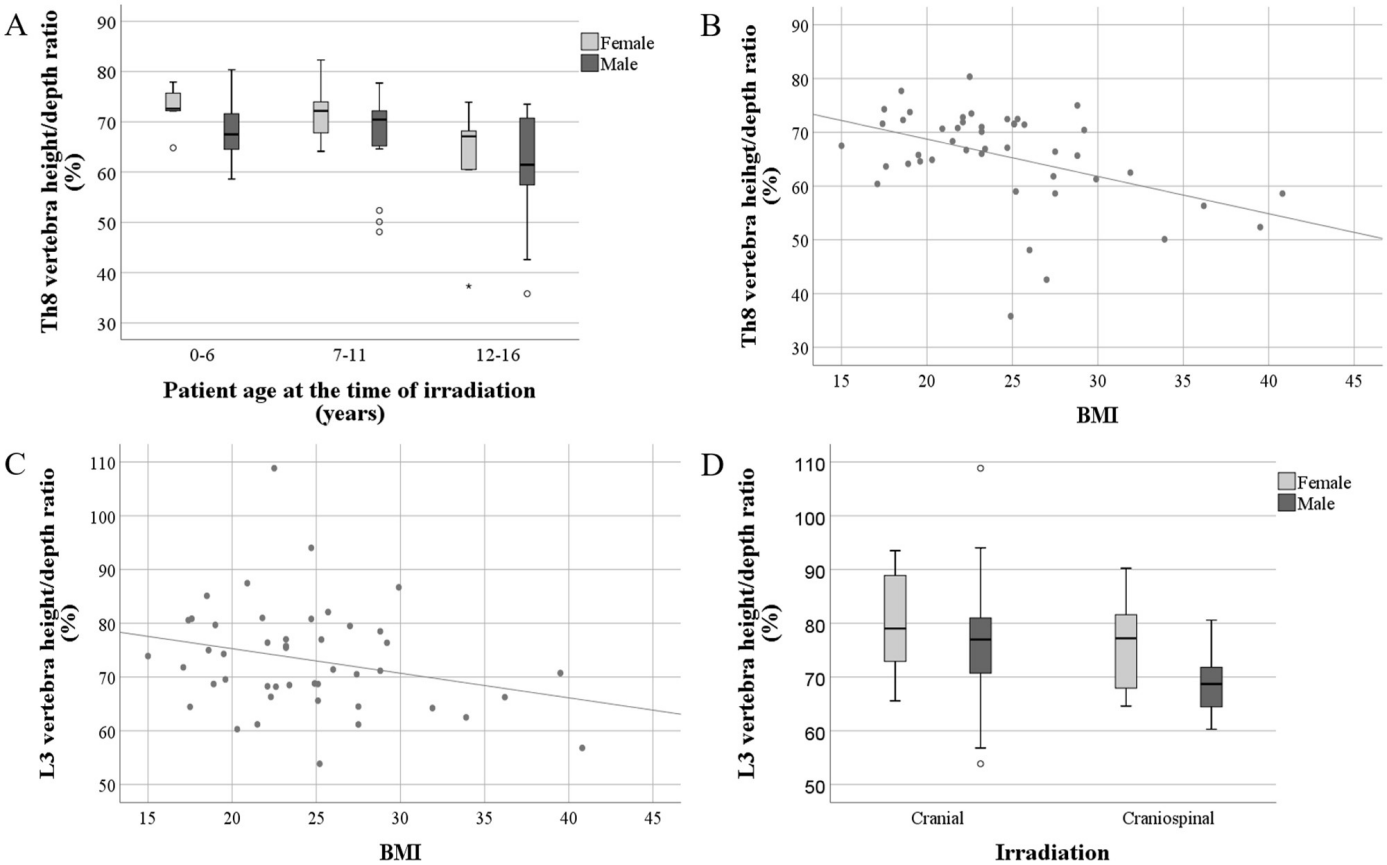
Association of the vertebral morphology to the BMI, mode of irradiation and treatment age. Association of the Th8 vertebra morphology with treatment age (**A**) and BMI (**B**). Association of the L3 vertebra morphology with BMI (**C**) and mode of irradiation (**D**).

Th8 height/depth ratio differed between the treatment age groups. A statistically significant difference was observed between the youngest and oldest age groups, [mean 69.8 vs 61.3, (mean difference (MD) 8.5, 95% confidence interval (CI) 2.4 to 14.7), *p* = 0.009]. Th8 height/depth ratio was the smallest in the oldest treatment age group (Fig 3A). BMI correlated negatively to the Th8 height/depth ratio in males (Fig 3B). Corticosteroid doses did not correlate to the Th8 height/depth ratio (*r* = -0.006, *p* = 0.962).

To evaluate if the area of the irradiation had an effect on vertebral morphology, Th8 height/depth ratio was compared between patients who had received cranial irradiation and patients who had received craniospinal irradiation. No statistically significant differences were found in Th8 height/depth ratio between these groups [mean 68.2 vs 65.9, (MD = 2.3, 95% CI -2.0 to 6.6), p = 0.287].

In multivariate analysis, BMI (adjusted β = -0.377, 95% CI (-0.678 to -0.076), *p* = 0.015), treatment age (adjusted β = -0.782, 95% CI (-1.248 to -0.316), *p* = 0.001) and patient gender (adjusted β = -5.984, 95% CI (-9.978 to -1.990), *p* = 0.004) associated significantly with the Th8 morphology (R^2^ adj = 0.233). When running multivariate regression analysis separately on males and females, the higher treatment age associated significantly with lower Th8 height/depth ratio in females (adjusted β = -0.902, 95% CI (-1.740 to -0.064), *p* = 0.036) (R^2^ adj = 0.147). In males, higher BMI (adjusted β = -0.638, 95% CI (-1.054 to -0.222), *p* = 0.003) and older treatment age (adjusted β = -0.688, 95% CI (-1.254 to -0.123), *p* = 0.018) associated with lower Th8 height/depth ratio (R^2^ adj = 0.259).

#### Morphology of the L3 vertebra

No correlation between height/depth ratio of L3 vertebra and patient treatment age was detected (*r* = -0.130). However, L3 height/depth ratios were smaller in patients who had received chemotherapy [mean 73.5 vs 78.5, (MD = 5.0, 95%CI 0.3 to 9.8), *p* = 0.038]. When females and males were analyzed separately, no statistically significant effect of chemotherapy was detected in females *(p* = 0.427), whereas in males a significant effect was observed [mean 70.6 vs 77.4, (MD 6.8, 95% CI 1.0 to 12.6), *p* = 0.023]. In males, BMI correlated negatively to the L3 height/depth ratio (Fig 3C), and there was a negative correlation between corticosteroid dose and L3 height/depth ratio (*r* = -0.333, *p* = 0.024).

When comparing the effects of the different areas of irradiation, L3 height/depth ratio was smaller in patients who had received craniospinal irradiation versus those who had received local, full cranial or stereotactic irradiation (71.0 vs 78.3 (MD = 7.3, 95% CI 2.9 to 11.8), *p* = 0.002) (Fig 3D). In females, no difference between the L3 height/depth ratio values in different irradiation groups was noted *(p* = 0.284). However, there was a significant difference between the groups in males [mean 68.8 vs 77.0, (MD 8.3, 95% CI 2.8 to 13.7), *p* = 0.004] (Fig 3D). Thereby, craniospinal irradiation associated with lower L3 height/depth ratio in males.

In multivariate regression analysis, patient gender (adjusted β = -4.857, 95% CI (-9.381 to -0.333), *p* = 0.036) and craniospinal irradiation (adjusted β = -6.829, 95% CI (-11.265 to -2.393), *p* = 0.003) associated with L3 vertebra morphology (R^2^ adj = 0.166). When comparing separately females and males, females did not have any statistically significant predictors for L3 vertebra morphology. In males, craniospinal irradiation (adjusted β = -8.377, 95% CI (-13.685 to -3.069), *p* = 0.003) and higher BMI (adjusted β = -0.475, 95% CI (-0.950 to 0.0), *p* = 0.050) associated with lower L3 height/depth ratio (R^2^ adj = 0.210), respectively.

### Bone mineral density

The results from our previously published DXA measurements [9] were compared with the spinal MRI findings in this study. The BMD measurement data were available for 72 subjects and in 70 of those, data were recorded from all five measurement points including both femoral necks, hip bones and lumbar spine (Fig 1). When the cases with the z-score below the expected range (z ≤ -2.0) in at least one of the measured areas were compared with the cases with z-scores of > -2.0, no statistically significant difference in Th8 or L3 height/depth ratio was detected (Table 4). Differences on the treatments between these groups are shown in Table 4.

**Table 4:** Tumor treatments, treatment age and vertebral height/depth ratios in subjects with z-score ≤ -2.0 and z-score > -2.0.

			z-score ≤ -2.0	z-score > -2.0	*p*-value
Females					
	Craniospinal irradiation, *n* (%)	yes	5 (62.5)	3 (37.5)	0.004
		no	1 (5.6)	17 (94.4)	
	Corticosteroid dose, median (IQR)		6.6 (7.4)	2.1 (3.3)	0.033
	Age at the irradiation, yr, mean (SD)		7.2 (4.5)	9.3 (3.9)	0.278
	Th8 vertebra height/depth ratio, mean (SD)		74.1 (5.9)	68.6 (8.9)	0.179
	L3 vertebra height/depth ratio, mean (SD)		81.4 (9.1)	78.9 (8.6)	0.547
Males					
	Craniospinal irradiation, *n* (%)	yes	3 (14.3)	18 (85.7)	0.188
		no	8 (32.0)	17 (68.0)	
	Corticosteroid dose, median (IQR)		1.5 (6.7)	1.4 (4.9)	0.894
	Age at the irradiation, yr, mean (SD)		5.5 (2.9)	8.8. (4.2)	0.017
	Th8 vertebra height/depth ratio, mean (SD)		67.3 (8.2)	65.4 (9.3)	0.548
	L3 vertebra height/depth ratio, mean (SD)		76.5 (13.2)	72.7 (8.4)	0.260

IQR = interquartile range, SD = standard deviation, yr = years, Th = thoracic, L = lumbar

### Final height

**Fig 4:**
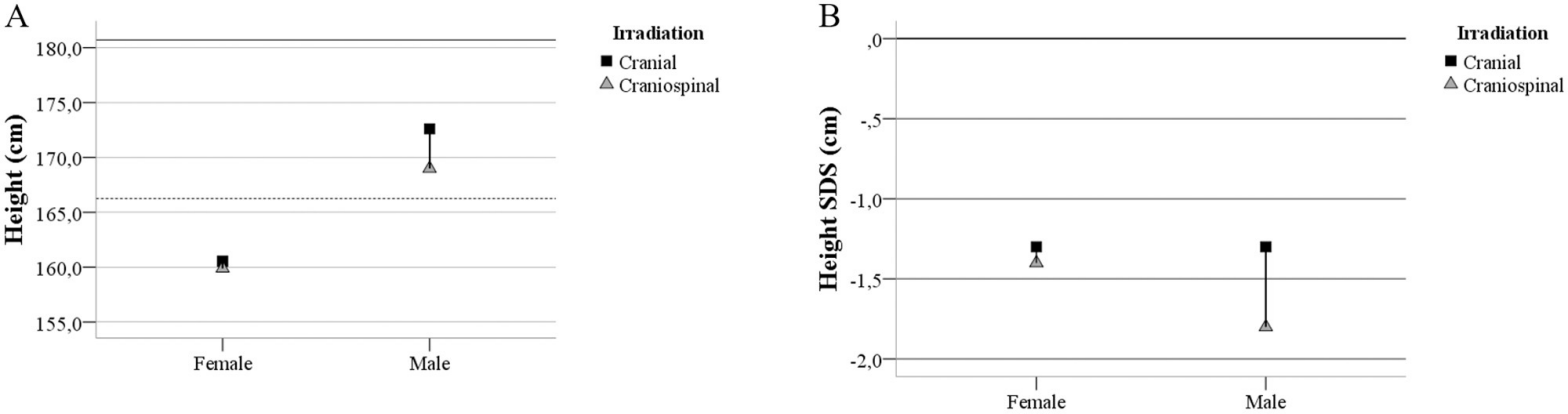
Comparison of the height of the study subjects with the general Finnish population. A: Height (cm) by gender, adult male height reference (solid line), adult female height reference (dotted line). B: Height standard deviation score (SDS) by gender with 0 SDS (dashed line). Cranial irradiation (solid square), craniospinal irradiation (triangle).

The effects of the different irradiation areas on final height were analyzed. Patients who had received craniospinal irradiation were shorter than those having undergone brain irradiation only, although the difference was statistically significant only in males (females *p* = 0.610 and males *p* = 0.047). Irrespective of irradiation type, subjects in this study were generally shorter compared to the general Finnish population, with the negative height SDS distribution in both gender subgroups (Fig 4, Table 5). Radiation area predicted height SDS in males so that the patients who received craniospinal irradiation tended to be shorter *(p* = 0.062). There was no significant correlation between Th8 or L3 height/depth ratio and subject height irrespective of gender.

**Table 5:** Height and height SDS of the subjects by irradiation type.

		Cranial	Craniospinal	*p*-value
Height (cm), mean/median (SD)				
	Female	157.5/160.6 (10.9)	159.4/159.9 (4.0)	0.610
	Male	172.9/172.6 (7.1)	168.3/169.0 (8.2)	0.047
Height SDS (unit), mean/median (SD)				
	Female	-1.9/-1.3 (2.0)	-1.5/-1.4 (0.7)	0.571
	Male	-1.1/-1.3 (1.3)	-1.9/-1.8 (1.3)	0.062

Standard deviation (SD), Standard deviation score (SDS)

## Discussion

Radiation therapy is an effective and often necessary treatment of malignant tumors and improves survival [4]. However, the long-term survivors of cancer suffer from serious side effects caused by ionizing radiation to surrounding tissues of the tumor [4,5,9,18]. In the present study, patients treated for brain tumors in childhood had vertebral deformities and fractures. Patients were shorter compared with the general population, suggesting a major impact on vertebral growth as also previously demonstrated [12,13].

Due to the absorbed dose of the irradiated bones, patients may have an increased risk for fractures [19]. BMD measured by DXA has an important role in the evaluation of osteoporosis, and low BMD may correlate with the increased risk of fractures [6]. Adult survivors of childhood lymphoblastic leukemia are reported to have low BMD, suggesting a failure to achieve normal peak bone mass during skeletal maturation [20]. Vertebral fragility fractures are the most common osteoporotic fractures, and they are usually asymptomatic incidental imaging findings [21]. In the previous study by Remes et al. concerning the same study population as in this study, low BMD z-scores were shown to be the common findings [9]. In this study, we report an undiagnosed vertebral fracture of 4.2% of the subjects. Only one subject had both a BMD z-score of ≤ -2.0 and vertebral fracture. The patient records of the three subjects with vertebral fractures revealed fractures of the long bones. Although Baxter et al. showed that radiation damage on bone is limited to the radiation field with no increase in fracture risk to nonirradiated bone [22], other factors, including brain surgery, local brain irradiation, chemotherapeutic agents and corticosteroids, are suggested to increase the risk for osteopenia and fractures [23].

The association of low BMD and long-bone fractures was noted in the survivors of irradiated pediatric brain tumors by Remes et al. [9]. The patients with childhood brain tumors were found to have a higher risk for vertebral fractures, but according to our findings, BMD is a weak predictor of vertebral fractures. Similarly, low BMD of the lumbar spine in children has not been associated with vertebral fractures [24]. Subjects with a BMD z-score of ≤ -2.0 did not have a significantly different Th8 or L3 vertebra height/depth ratio compared with subjects with a z-score of > -2.0. Despite the fact that BMD did not correlate with the vertebral morphology, this finding does not rule out the possibility that BMD may be one of the underlying factors predisposing to the vertebral fractures and changes in vertebral morphology. Although children with corticosteroid-treated illnesses are known to be at risk for osteoporotic vertebral fractures [25], corticosteroids did not have a significant influence on the vertebral morphology in this study. The lack of a significant effect of corticosteroids on the vertebral morphology might be due to the fact that almost all the patients (90%) were treated with corticosteroids. Steroid toxicity has been reported to be associated with oxidative stress contributing to morphological changes on bone and increased fracture risk [26]. Thus, a mismatch between BMD data and bone morphology in patients treated with steroids is suggested to be attributed to a complex mechanism of bone health [27]. Interestingly, compressed vertebral bodies in children with acute lymphoblastic leukemia have been shown to remodel during follow-up to achieve normal size and shape in those with sufficient residual spinal growth left [28].

Late endocrine effects, including reduced bone growth and density following radiation and chemotherapy, are demonstrated among adult survivors of pediatric brain tumors [5,29]. Cranial or craniospinal irradiation affecting the hypothalamic-pituitary axis can result in GH deficiency, which decreases final height [5,12,29,30]. In addition, radiation may cause direct growth disturbances on the developing skeleton in children for any given dose, and the effects are greater with higher doses and those irradiated at a young age [12]. Irreversible morphological changes, including vertebral scalloping, hypoplasia and end-plate irregularities as well as growth arrest of the spine associated with decreased vertebral height, are demonstrated to be characteristic radiation-induced effects [18,31]. In the study subjects, there was at least one grade 1 or higher vertebra according to Genant’s visual SQ method among six males and one female. Three of the seven of these subjects had a vertebral fracture, and four of them had several adjacent flat vertebrae with end-plate irregularities, suggesting radiation-induced growth disturbance of the spine. The biconcave vertebrae with central endplate depression of these four irradiated subjects resembled those of the vertebrae of patients suffering from sickle cell disease or thalassemia. Irradiation may induce a similar mechanism of microvascular endplate occlusion leading to infarcts and osteonecrosis, and subsequent overgrowth of the surrounding portions of the endplate. [25,31]. In addition to sickle cell disease or thalassemia, the biconcave vertebral structure, known as H- or fish-shaped vertebra, is a usual finding of vertebral fracture caused by low BMD [14].

Irradiation-induced direct damage to vertebrae in combination with other treatment-induced late effects may cause osteoporosis, low-energy fractures, pain and other skeletal problems that affect the quality of life of the brain tumor survivors during adulthood. To identify the late sequelae of the childhood tumor treatments, survivors need regular follow-up in health care. Asymptomatic vertebral fracture in the absence of major trauma indicates bone fragility, and intervention for proper nutrition with adequate calcium and vitamin D intake, guidance for regular physical activity or targeted medication may be needed [32].

Boys seem to be more prone to growth impairment due to irradiation, probably because they have a greater percentage of spinal growth remaining compared with girls [33]. In this study, males tended to have a lower height/depth ratio in both Th8 and L3 vertebrae than females. Th8 height/depth ratio seemed to be lower in patients who had undergone radiation therapy in late childhood (12–16 years) than those treated younger. In a recent study of children with radiation-treated paravertebral neuroblastoma, higher doses, older treatment age, male gender and thoracic location were associated with decreased vertebral body growth [34]. Thus, the vertebrae of males may be more sensitive to irradiation.

BMI was a significant predictor of the Th8 vertebra morphology. More specifically, in males BMI was the most significant predictor of the Th8 vertebra morphology when using multivariate regression analysis. Overweight male subjects tended to have the lowest Th8 height/depth ratio. BMI also correlated to L3 height/depth ratio, but correlation was not as strong and significant as in Th8 vertebra. BMI has been demonstrated to correlate negatively to BMD, especially in females in this same patient cohort [9]. Against the traditional paradigm of the relationship of BMI and BMD, these results are consistent with the previous study reporting that obesity is negatively associated with BMD [35]. In addition, body fat mass has been demonstrated to have inverse effects on parameters related to the structure and strength of the bone in young adults, suggesting that obesity may not be beneficial to bone health [36]. Obesity-induced inflammation plays a crucial role in bone metabolism [37]. Inflammation may distract the normal recovery process of the bone tissue after irradiation, predisposing subjects to fractures and bone morphology changes.

The L3 height/depth ratio was lower in males who had received craniospinal irradiation or chemotherapy. Chemotherapy and craniospinal irradiation are suggested to have a combined effect on the morphology of the L3 vertebra. In the multivariate linear regression, craniospinal irradiation predicted L3 morphology more than chemotherapy. In childhood brain tumor survivors treated by irradiation, the prevalence of obesity and pituitary hormone deficits have been reported to be associated with lowered height SDS [38]. Both standing and sitting height have been decreased after entire spine irradiation before puberty in a study of Hodgkin lymphoma survivors [39]. Growth data analysis of the patients in this study showed that irradiation of the spine affected total body height, which was statistically significant in males. Consequently, the patients who received craniospinal irradiation tended to be shorter than the general population. Craniospinal irradiation has been shown to have a more pronounced effect on spinal growth than cranial irradiation, and the growth impairment is greater in younger children [12]. This indicates that irradiation of the spinal structures may result in vertebral changes and a growth deficit [13].

## Conclusion

In this study, we report a high number of vertebral abnormalities in long-term survivors of irradiated pediatric brain tumors. We found evidence that craniospinal irradiation, along with other treatment- and patient-related factors, may have long-term effects on vertebral shape and morphology as well as final height. The impact of our findings on long-term skeletal health calls for further studies.

## Supporting information

S1 Dataset The values used for statistical analyses. (PDF)
