# Supplementary material for: Late vertebral side effects in long-term survivors of irradiated childhood brain tumor
Source: PLoS One. 2018 Dec 18;13(12):e0209193. doi: 10.1371/journal.pone.0209193 (PMC6298650; doi:10.1371/journal.pone.0209193)
Supplement: S1 Dataset — (PDF) [file pone.0209193.s001.pdf]

	Height/depth ratio				
SDI	Th8	L3	Age at the study MR	Chemotherapy (1=yes, 0=no)	Spinal irradiation (1=yes, 0=no)
0	71,6	68,7	24	1	1
0,5	58,6	56,8	29	0	0
2,5	35,8	68,8	31	1	1
5	42,6	79,5	33	0	0
1	60,4	71,8	18	1	1
1	73,5	68,2	43	1	0
0	75	78,5	28	0	0
0	66,7	66,3	30	1	0
2	48,1	71,4	41	0	0
0	66,9	88,9	33	0	0
0	72,6	72,9	28	1	0
3	37,3	67	43	1	1
0,5	66,9	68,5	27	1	0
0	66,4	64,5	19	1	1
0	72,8	76,4	28	1	0
0	77,9	93,5	32	1	0
0	67,5	73,9	21	1	0
1	50,1	62,5	30	1	1
0	72,2	84,5	27	0	0
0	72,3	75	35	1	1
0,5	72,2	67,7	39	1	0
0	71,5	65,6	26	1	1
0	72,2	73,5	31	1	0
0	73,9	90,9	37	0	0
0	67,3	77,1	39	0	0
0	70,11	75,78	33	0	0
0	75	79,38	25	1	1
0	60,5	72,67	38	0	0
0	72,17	83,91	26	1	0
0	67,13	80,8	27	0	0
0	70,8	81	36	0	0
0	82,29	83,14	18	1	1

0	65,66	71,17	27	1	1
0,5	58,62	61,17	21	1	1
0	73,76	79,69	23	1	1
0	71,89	68,28	26	1	1
0	65,8	81,61	26	1	1
0	72,46	94,03	24	0	0
0,5	68,18	65,56	37	1	0
0	70,42	76,37	29	0	1
1	61,29	86,69	27	0	0
0,5	70,99	75,45	28	0	0
0	64,14	68,7	26	1	1
0	62,5	64,23	28	0	1
0	64,13	67,95	21	1	1
0	66,02	77,02	32	1	0
0	76,19	92,09	24	1	0
0,5	64,89	60,3	24	1	1
0	75,71	64,61	19	1	1
0	52,36	70,73	29	0	0
0	80,35	108,84	24	0	0
0	80,62	92,56	30	1	0
0	64,83	74,88	20	1	0
4,5	56,32	66,25	19	1	1
0	71,43	82,08	31	1	0
0	70,45	82,41	23	0	0
0	72,48	76,97	36	0	0
0	61,81	70,52	25	1	1
0	68,33	61,19	20	0	1
0	74,3	64,45	25	1	1
0	64,6	69,55	18	0	0
0	77,71	85,1	16	0	0
0	71,59	80,58	16	1	1
0	63,65	80,85	23	1	0
0	66,67	72,78	32	0	0
0	73,39	77,2	24	1	1

0,5	59	53,86	34	1	0
0	74,56	75,4	34	1	1
0	65,78	74,3	19	1	1
0	70,67	87,46	24	1	0
0,5	68,93	79,01	40	0	0
0	72,17	90,23	19	1	1
				0	0
				1	0

				Z-score		
**Corticosteroid dose (g/m²)**	**Sitting height (cm)**	**Height (cm)**	**BMI**	**Right femur BMD**	**Left femur BMD**	**Right hip BMD**
24	68,6	168,5	25,1	-0,9	0,1	-0,5
1,02	98,3	196,9	40,8			
3,77	86,7	180,9	24,9	-0,8	-0,4	-0,6
0,93	82,7	166,1	27	-1	-1,3	-0,8
1,42	85,2	169,4	17,1	-0,4	-0,6	-1,1
1,72	89,9	183,3	22,6	-1,6	-1,6	-1,5
2,68	84	169,1	28,8	-0,4	-0,8	-0,6
6,77	84,6	172,6	22,3	-1,7	-2,5	-1,8
7,98	82,3	173,5	26	-2,9	-3,4	-3,3
0,84	85,7	168,2	35	-0,8	-0,7	-1,1
17,25	72,6	147,2	21,8	-1,7	-1,4	-1,5
2,28	77,5	157,8	21,9	-1,2	-1,9	-1
3,05	82,3	176,2	23,4	-1,4	-0,1	-0,9
9,03	78,7	163,2	27,5	-0,7	-1,1	-1,2
0	80,5	164,2	22,1	-2,5	-2,4	-2,2
0,52	77,3	150,7	21,4	0,2	0,2	0,8
5,52	81,2	164,3	15	-0,4	-1	-0,1
1,64	83,3	173,4	33,9	-1,3	-1,4	-0,7
1,98	78,2	146,4	32,4	-0,6	-0,4	0
0,93	89	183,9	18,6	-1,1	-1	-0,9
0		142	11,5	-0,7	-1,2	-0,4
6,4	73,5	148,4	25,1	-2,6	-3,3	-2,5
3,68	81	157,9	30,3	-1,6	-1,2	0,1
0,48	78,8	152,9	27,6	0,1	0,5	0,5
1,49	85,5	166,3	25,7	-0,3	-0,1	-0,9
1,55	86,2	169,2	23,2	-1,8	-1,4	-1
5,68	75,5	153,7	29,2	-2	-1,9	-2,1
0,62	88,6	165	35,4	0,8	0,6	1,3
7,31	88	148,4	34,8	0,4	-0,2	1,1
1,49	89,7	168,4	24,7	-1,8		-1,5
0	93,4	181,2	21,8	-0,5	0,1	-0,3
8,15	80,6	160,8	18	-1,1	-1	-1,4

11,4	95,1	177,2	28,8	-1,6	-1,6	-1,3
17,56	84,7	174,5	27,5	-0,8	-0,7	-0,3
12,75	81,6	163	19	-1,3	-1,2	-1,1
7,16	83,2	159,5	22,1	-0,2	-0,1	-0,1
3,23	75,1	156,2	29,8	-1,5	0	-1,3
0	89,1	168,3	24,7	-0,6	-0,1	0,2
2,5	88	163,7	26,3	0,2	0,3	1,1
1,01	89,1	169	29,2	-0,4	0,2	-0,6
0	91,1	173	29,9	-1,7	-1,1	-2
0,12	94,2	176,6	23,2	-2,5	-2,3	-2
8,17	88,8	173,6	18,9	-0,8	-0,6	-0,5
0,85	90,9	173,5	31,9	-0,2	0	-0,1
3,52	81,4	159,9	20,7	-1,9	-1,9	-2
4,23	92,4	174,8	23,2	-1	-1	-1
17,38		136	19	-2,5	-1,7	-3,3
17,53	74,7	160,2	20,3	0	-0,4	-1
0,56	84,5	156,1	40,1			
0	97	174,3	39,5	0,9	0,9	0,8
2,52	90,9	170,2	22,5	-0,9	-1	-0,7
0,58	87,4	168,2	27,3	0,6	0,6	0,3
4,29	87,4	174	30,8	0,4	0,7	-0,1
0,22	85,1	170,8	36,2	-0,2	0,7	0,6
3,63	91	171,7	25,7	-1,7	-1,1	-1,4
0,2	89	169,9	27,1	1	1,6	1,1
0,1	93,8	172,6	25,3	-0,6	-0,9	-1
11,25	82,7	167,6	27,4	-0,5	-0,9	-0,2
1,06	84,4	163,9	21,5	-1,9	-1,8	-1,6
1,12	87	169,8	17,5	-1,3	-1,9	-1,4
1,27	87,8	166,4	19,6	-2,4	-2,8	-2
0,13	86,8	165,9	18,5	-1	-0,5	-1,1
1,46	74,5	157,2	17,4	-2,6	-3,1	-3,1
0,65	77	165,2	17,6	-0,9	-1,2	-1
	88,7	163,2	35	-0,2	-0,3	0,2
2,76	81,7	166,4	25	0,5	0,1	0,1

0	91,2	174,2	25,2		-0,7	
7,5	78,9	159,9	19,3	-1,3	-1,7	-1,3
0,88	81,3	166,5	19,5	0,4	0,4	0,2
0,38	94,6	180,3	20,9	-0,4	-0,3	-0,1
		151,1	43,5	0,6	0,8	2,3
1,87	87,1	164,1	17,4	-1,8	-1,4	-2,4
1,24	86,5	176,2	22,5	-1,2	-0,7	-0,9
12	88,5	163,5	28,2	-1	-0,9	-0,8

Left hip BMD	Lumbar spine BMD	Vertebral fracture (1=yes)	Gender (1=male, 0=female)	Treatment age	Diagnosis age	Th4
0	-1,3	0	1	3	3	0,0
		0	1	12	11	0,0
-0,8	0,7	0	1	15	15	0,0
-0,6	0,7	0	1	15	15	0,0
-1,1	-0,7	0	1	12	12	0,0
-1,5	-1,6	1	1	15	15	1,0
-0,8	1	0	1	4	4	0,0
-2,2	-1,8	0	1	5	5	0,0
-3,6	-3,9	1	1	11	11	0,5
-0,8	-1,5	0	0	15	15	0,0
-1,3	-0,9	0	0	4	4	0,0
-1,7	-0,3	1	0	14	14	0,0
-0,4	-0,6	0	1	12	12	0,0
-1,2	-1,4	0	1	8	8	0,0
-1,9	-1,2	0	1	2	2	0,0
0,8	0,5	0	0	4	4	0,0
-0,2	0,2	0	1	6	5	0,0
-0,6	-1,4	0	1	9	8	0,0
0,3	0,8	0	0	6	6	0,0
-1	-0,7	0	1	15	15	0,0
-0,4	0,9	0	0	8	8	0,0
-3	-0,2	0	1	5	4	0,0
0,3	0,3	0	0	5	5	0,0
0,5	0,4	0	0	15	15	0,0
-0,4	-0,2	0	0	15	15	0,0
-1	-1	0	1	5	5	0,0
-2,4	-1,1	0	0	2	2	0,0
1,4	0,9	0	0	12	12	0,0
1,1	0,4	0	0	5	4	0,0
	-2	0	1	4	4	0,0
0	0,1	0	1	14	14	0,0
-1,3	-2,4	0	0	8	8	0,0

-1,3	-1,1	0	1	5	5	0,0
-0,4	-0,6	0	1	1	1	0,0
-1,5	-1,7	0	1	4	4	0,0
-0,1	-0,6	0	1	10	10	0,0
-1,1	-0,9	0	0	10	10	0,0
0,4	-1	0	1	8	6	0,0
1,5	0,6	0	0	13	13	0,0
-0,1	1	0	1	8	8	0,0
-0,8	-1,7	0	1	5	5	0,0
-2,1	-1,4	0	1	7	4	0,0
-0,6	-0,3	0	1	4	4	0,0
-0,1	-0,4	0	1	12	11	0,0
-2	-2,1	0	0	11	11	0,0
-1,1	-1	0	1	9	9	0,0
-2,6	-2,6	0	0	1	1	0,0
-0,9	-2,4	0	1	6	6	0,0
		0	0	6	6	0,0
0,9	-0,9	0	1	8	8	0,0
-0,3	-2,1	0	1	2	1	0,0
0,7	-1	0	0	11	10	0,0
0,1	-0,4	0	0	5	1	0,0
0,8	-0,6	0	1	14	14	0,0
-1	-1,3	0	1	11	11	0,0
1,3	0,6	0	0	9	8	0,0
-0,9	-0,9	0	1	8	6	0,0
0	-0,1	0	1	2	2	0,0
-1,6	-1,5	0	1	3	3	0,0
-1,9	-0,7	0	1	10	10	0,0
-2,3	-3	0	1	10	9	0,0
-0,7	-1,1	0	1	7	4	0,0
-3,7	-4,2	0	1	3	1	0,0
-1,2	-1,8	0	1	5	5	0,0
0,5	0,8	0	0	11	10	0,0
0	0,1	0	0	10	10	0,0

0,1	-0,6	0	1	13	13	0,0
-1,7	-2,1	0	0	11	11	0,0
0,2	-1,5	0	1	11	11	0,0
0	-1,2	0	1	13	13	0,0
1,8	0,8	0	0	7	4	0,0
-2,2	-2,9	0	0	10	10	0,0
-0,7	0,4		1	10	8	
-0,5	-0,8		0	6	5	

Genant classification of vertebral fractures												
**Th5**	**Th6**	**Th7**	**Th8**	**Th9**	**Th10**	**Th11**	**Th12**	**L1**	**L2**	**L3**	**L4**	**Female Height**
0,0	0,0	0,0	0,0	0,0	0,0	0,0	0,0	0,0	0,0	0,0	0,0	168,2
0,0	0,0	0,5	0,0	0,0	0,0	0,0	0,0	0,0	0,0	0,0	0,0	147,2
0,0	0,0	1,0	0,5	0,5	0,5	0,0	0,0	0,0	0,0	0,0	0,0	157,8
1,0	1,0	1,0	1,0	1,0	0,0	0,0	0,0	0,0	0,0	0,0	0,0	150,7
0,0	0,0	0,0	0,0	0,0	0,0	0,5	0,5	0,0	0,0	0,0	0,0	146,4
0,0	0,0	0,0	0,0	0,0	0,0	0,0	0,0	0,0	0,0	0,0	0,0	142
0,0	0,0	0,0	0,0	0,0	0,0	0,0	0,0	0,0	0,0	0,0	0,0	157,9
0,0	0,0	0,0	0,0	0,0	0,0	0,0	0,0	0,0	0,0	0,0	0,0	152,9
0,5	0,0	0,0	0,0	0,0	0,0	0,0	0,0	1,0	0,0	0,0	0,0	166,3
0,0	0,0	0,0	0,0	0,0	0,0	0,0	0,0	0,0	0,0	0,0	0,0	153,7
0,0	0,0	0,0	0,0	0,0	0,0	0,0	0,0	0,0	0,0	0,0	0,0	165
0,0	1,0	0,0	2,0	0,0	0,0	0,0	0,0	0,0	0,0	0,0	0,0	148,4
0,0	0,0	0,0	0,0	0,0	0,0	0,0	0,0	0,0	0,0	0,5	0,0	160,8
0,0	0,0	0,0	0,0	0,0	0,0	0,0	0,0	0,0	0,0	0,0	0,0	156,2
0,0	0,0	0,0	0,0	0,0	0,0	0,0	0,0	0,0	0,0	0,0	0,0	163,7
0,0	0,0	0,0	0,0	0,0	0,0	0,0	0,0	0,0	0,0	0,0	0,0	163,5
0,0	0,0	0,0	0,0	0,0	0,0	0,0	0,0	0,0	0,0	0,0	0,0	159,9
0,0	0,0	0,0	0,0	0,0	0,0	0,0	1,0	0,0	0,0	0,0	0,0	136
0,0	0,0	0,0	0,0	0,0	0,0	0,0	0,0	0,0	0,0	0,0	0,0	156,1
0,0	0,0	0,0	0,0	0,0	0,0	0,0	0,0	0,0	0,0	0,0	0,0	168,2
0,0	0,0	0,0	0,0	0,0	0,5	0,0	0,0	0,0	0,0	0,0	0,0	174
0,0	0,0	0,0	0,0	0,0	0,0	0,0	0,0	0,0	0,0	0,0	0,0	169,9
0,0	0,0	0,0	0,0	0,0	0,0	0,0	0,0	0,0	0,0	0,0	0,0	163,2
0,0	0,0	0,0	0,0	0,0	0,0	0,0	0,0	0,0	0,0	0,0	0,0	166,4
0,0	0,0	0,0	0,0	0,0	0,0	0,0	0,0	0,0	0,0	0,0	0,0	159,9
0,0	0,0	0,0	0,0	0,0	0,0	0,0	0,0	0,0	0,0	0,0	0,0	151,1
0,0	0,0	0,0	0,0	0,0	0,0	0,0	0,0	0,0	0,0	0,0	0,0	164,1
0,0	0,0	0,0	0,0	0,0	0,0	0,0	0,0	0,0	0,0	0,0	0,0	
0,0	0,0	0,0	0,0	0,0	0,0	0,0	0,0	0,0	0,0	0,0	0,0	
0,0	0,0	0,0	0,0	0,0	0,0	0,0	0,0	0,0	0,0	0,0	0,0	
0,0	0,0	0,0	0,0	0,0	0,0	0,0	0,0	0,0	0,0	0,0	0,0	
0,0	0,0	0,0	0,0	0,0	0,0	0,0	0,0	0,0	0,0	0,0	0,0	

0,0	0,0	0,0	0,0	0,0	0,0	0,0	0,0	0,0	0,0	0,0	0,0	
0,0	0,0	0,0	0,0	0,0	0,5	0,0	0,0	0,0	0,0	0,0	0,0	
0,0	0,0	0,0	0,0	0,0	0,0	0,0	0,0	0,0	0,0	0,0	0,0	
0,0	0,0	0,0	0,0	0,0	0,0	0,0	0,0	0,0	0,0	0,0	0,0	
0,0	0,0	0,0	0,0	0,0	0,0	0,0	0,0	0,0	0,0	0,0	0,0	
0,0	0,0	0,0	0,0	0,0	0,0	0,0	0,0	0,0	0,0	0,0	0,0	
0,0	0,0	0,0	0,0	0,0	0,0	0,0	0,0	0,0	0,0	0,0	0,5	
0,0	0,0	0,0	0,0	0,0	0,0	0,0	0,0	0,0	0,0	0,0	0,0	
0,0	0,0	1,0	0,0	0,0	0,0	0,0	0,0	0,0	0,0	0,0	0,0	
0,0	0,0	0,5	0,0	0,0	0,0	0,0	0,0	0,0	0,0	0,0	0,0	
0,0	0,0	0,0	0,0	0,0	0,0	0,0	0,0	0,0	0,0	0,0	0,0	
0,0	0,0	0,0	0,0	0,0	0,0	0,0	0,0	0,0	0,0	0,0	0,0	
0,0	0,0	0,0	0,0	0,0	0,0	0,0	0,0	0,0	0,0	0,0	0,0	
0,0	0,0	0,0	0,0	0,0	0,0	0,0	0,0	0,0	0,0	0,0	0,0	
0,0	0,0	0,0	0,0	0,0	0,0	0,0	0,0	0,0	0,0	0,0	0,0	
0,5	0,0	0,0	0,0	0,0	0,0	0,0	0,0	0,0	0,0	0,0	0,0	
0,0	0,0	0,0	0,0	0,0	0,0	0,0	0,0	0,0	0,0	0,0	0,0	
0,0	0,0	0,0	0,0	0,0	0,0	0,0	0,0	0,0	0,0	0,0	0,0	
0,0	0,0	0,0	0,0	0,0	0,0	0,0	0,0	0,0	0,0	0,0	0,0	
0,0	0,0	0,0	0,0	0,0	0,0	0,0	0,0	0,0	0,0	0,0	0,0	
0,0	0,0	0,0	0,0	0,0	0,0	0,0	0,0	0,0	0,0	0,0	0,0	
0,0	0,0	0,0	0,0	0,0	0,0	0,5	0,0	1,0	1,0	1,0	1,0	
0,0	0,0	0,0	0,0	0,0	0,0	0,0	0,0	0,0	0,0	0,0	0,0	
0,0	0,0	0,0	0,0	0,0	0,0	0,0	0,0	0,0	0,0	0,0	0,0	
0,0	0,0	0,0	0,0	0,0	0,0	0,0	0,0	0,0	0,0	0,0	0,0	
0,0	0,0	0,0	0,0	0,0	0,0	0,0	0,0	0,0	0,0	0,0	0,0	
0,0	0,0	0,0	0,0	0,0	0,0	0,0	0,0	0,0	0,0	0,0	0,0	
0,0	0,0	0,0	0,0	0,0	0,0	0,0	0,0	0,0	0,0	0,0	0,0	
0,0	0,0	0,0	0,0	0,0	0,0	0,0	0,0	0,0	0,0	0,0	0,0	
0,0	0,0	0,0	0,0	0,0	0,0	0,0	0,0	0,0	0,0	0,0	0,0	
0,0	0,0	0,0	0,0	0,0	0,0	0,0	0,0	0,0	0,0	0,0	0,0	
0,0	0,0	0,0	0,0	0,0	0,0	0,0	0,0	0,0	0,0	0,0	0,0	
0,0	0,0	0,0	0,0	0,0	0,0	0,0	0,0	0,0	0,0	0,0	0,0	
0,0	0,0	0,0	0,0	0,0	0,0	0,0	0,0	0,0	0,0	0,0	0,0	

0,0	0,0	0,0	0,0	0,0	0,0	0,0	0,0	0,0	0,0	0,0	0,5	
0,0	0,0	0,0	0,0	0,0	0,0	0,0	0,0	0,0	0,0	0,0	0,0	
0,0	0,0	0,0	0,0	0,0	0,0	0,0	0,0	0,0	0,0	0,0	0,0	
0,0	0,0	0,0	0,0	0,0	0,0	0,0	0,0	0,0	0,0	0,0	0,0	
0,0	0,0	0,0	0,0	0,0	0,0	0,0	0,0	0,0	0,0	0,5	0,0	
0,0	0,0	0,0	0,0	0,0	0,0	0,0	0,0	0,0	0,0	0,0	0,0	

Female Height SDS	Male Height	Male Height SDS
0,1	168,5	-2
-3,8	196,9	2,6
-1,8	180,9	0
-3,1	166,1	-2,4
-3,9	169,4	-1,7
-4,7	183,3	0,4
-1,8	169,1	-1,9
-2,7	172,6	-1,3
-0,2	173,5	-1,2
-2,5	176,2	-0,7
-0,5	163,2	-2,8
-3,5	164,2	-2,7
-1,1	164,3	-2,7
-2,1	173,4	-1,2
-0,7	183,9	0,5
-0,7	148,4	-5,3
-1,4	176,2	-0,7
-5,8	169,2	-1,8
-2	168,4	-2
0,1	181,2	0,1
1,2	177,2	-0,6
0,4	174,5	-1
-0,8	163	-2,9
-0,2	159,5	-3,5
-1,4	168,3	-2
-3	169	-1,9
-0,6	173	-1,3
	176,6	-0,7
	173,6	-1,2
	173,5	-1,2
	174,8	-1
	160,2	-3,3

	174,3	-1
	170,2	-1,7
	170,8	-1,6
	171,7	-1,5
	172,6	-1,3
	167,6	-2,1
	163,9	-2,7
	169,8	-1,8
	166,4	-2,1
	165,9	-1,5
	157,2	-3,1
	165,2	-2,5
	174,2	-1,1
	166,5	-0,2
	180,3	2,4
